# Genetic Mapping and Analysis of a Compact Plant Architecture and Precocious Mutant in Upland Cotton

**DOI:** 10.3390/plants11111483

**Published:** 2022-05-31

**Authors:** Lei Chao, Zhenyuan Pan, Jing Wang, Yuanlong Wu, Guangling Shui, Nurimanguli Aini, Binghui Tang, Chunping Guo, Peng Han, Panxia Shao, Xiaomin Tian, Xinyi Chang, Qiushuang An, Chunmei Ma, Chunyuan You, Longfu Zhu, Xinhui Nie

**Affiliations:** 1 Key Laboratory of Oasis Ecology Agricultural of Xinjiang Production and Construction Corps, Agricultural College, Shihezi University, Shihezi 832003, China; clshzu@163.com (L.C.); panzhenyuandawood@163.com (Z.P.); shuiguangling2022@163.com (G.S.); gulishzu@163.com (N.A.); guochunping@stu.shzu.edu.cn (C.G.); han_peng@stu.shzu.edu.cn (P.H.); shaopanxia@stu.shzu.edu.cn (P.S.); tianxiaomin@stu.shzu.edu.cn (X.T.); cxy_shzu@126.com (X.C.); an17590932801@163.com (Q.A.); 13009683726@163.com (C.M.); 2 College of Informatics, Huazhong Agricultural University, Wuhan 430070, China; silentj@webmail.hzau.edu.cn; 3 National Key Laboratory of Crop Genetic Improvement, College of Plant Sciences & Technology, Huazhong Agricultural University, Wuhan 430070, China; wyl19880322@163.com; 4 Cotton Research Institute of the Shihezi Academy of Agriculture Science, Shihezi 832011, China; ttangbbing@aliyun.com

**Keywords:** upland cotton, nulliplex branch, early maturity, *Ghnb5*, genetic mapping

## Abstract

With the promotion and popularization of machine cotton-picking, more and more attention has been paid to the selection of early-maturity varieties with compact plant architecture. The type of fruit branch is one of the most important factors affecting plant architecture and early maturity of cotton. Heredity analysis of the cotton fruit branch is beneficial to the breeding of machine-picked cotton. Phenotype analysis showed that the types of fruit branches in cotton are controlled by a single recessive gene. Using an F_2_ population crossed with Huaxin102 (normal branch) and 04N-11 (nulliplex branch), BSA (Bulked Segregant Analysis) resequencing analysis and *GhNB* gene cloning in 04N-11, and allelic testing, showed that fruit branch type was controlled by the *GhNB* gene, located on chromosome D07. *Ghnb5*, a new recessive genotype of *GhNB*, was found in 04N-11. Through candidate gene association analysis, SNP 20_15811516_SNV was found to be associated with plant architecture and early maturity in the Xinjiang natural population. The *GhNB* gene, which is related to early maturity and the plant architecture of cotton, is a branch-type gene of cotton. The 20_15811516_SNV marker, obtained from the Xinjiang natural population, was used for the assisted breeding of machine-picked cotton varieties.

## 1. Introduction

With the popularization of mechanized cotton harvesting, the selection and breeding of early-maturity varieties with compact plant architecture, concentrated flowering and early maturity are highlighted. The plant architecture of cotton is determined by the fruit branch type, internode length and fruit branch angle. Among them, the fruit branch type is the most influential factor. The study of fruit branch type is helpful in the selection and breeding of machine-harvested cotton. Xinjiang is China’s main cotton planting area, where cotton yield and quality are higher than the national average level. In 2020, Xinjiang’s cotton planting area was 2,501,900 hectares, and the output of cotton was 5.16 million tons [1]. The cotton output of Xinjiang accounts for 87.3% of the total national cotton output [1]. Due to the improvement in cotton quality in Xinjiang, the self-sufficiency rate of high-quality raw cotton in China has been constantly improving; from 2016 to 2018, the self-sufficiency rate of high-quality raw cotton increased from 30% to 60% [2]. In 2019, the output value of the cotton industry reached 70 billion, and about one million people were engaged in cotton-related jobs [3]. The healthy development of the cotton industry in Xinjiang influences the stability of the Xinjiang economy and the supply of cotton in China and around the world.

In recent years, with the continuous improvement in residents’ incomes, the labor cost of cotton production has been increasing. The labor input is the biggest cost in traditional cotton production. The mechanization of planting and harvesting can improve production efficiency, and can reduce the cost of labor and the production cost of cotton. The plant architecture of cotton not only affects the ventilation and light transmission of the population, but also impacts mechanized harvesting [4]. The main stem and the vegetative branch of cotton display monopodial growth with an obvious main stem. The fruit branch of cotton displays sympodial growth, without an obvious main stem. The axial primordium gradually transitions from vegetative growth to reproductive growth, and develops into flowers. The primordium in the leaf axil below the apical branch develops vegetative growth to form the next fruit node [5]. Cotton has the capability for of unlimited growth, and is a perennial crop. Under suitable environmental conditions, the main stem, vegetative branches and fruit branches continue to grow, eventually forming a tower structure [6]. The loose plant architecture and long fruit branches or vegetative branches in the middle and lower part, which are not conducive to mechanized harvesting and rational dense planting, were found in the traditional cotton varieties. On the contrary, cotton varieties with compact plant architecture, densification resistance and a small fruit branch angle are more suitable for mechanized harvesting, and are conducive to yield increase via increasing planting density [7]. After cotton flocculation, leaf shedding, which can reduce the impurity rate and the pollution of seed cotton, have been performed using a defoliating agent [8]. In summary, compact plant architecture, flocculation concentration and defoliant-sensitive varieties are more suitable for mechanized harvesting.

The type of cotton fruit branch can be categorized as a nulliplex branch, determinate branch or normal branch, according to the number of fruit nodes. Cotton with the nulliplex branch has no obvious fruit branches, with a typical tubular structure; it has mostly early-maturity varieties of plant architecture, and the bolls grow directly in the axils of the main stem [5]. Cotton with the determinate branch has only one fruit node, and bolls that cluster at the top of fruit branch. Cotton with the normal branch has more than one fruit node, and bolls that are borne on the top of the fruit nodes [5]. In the plant architecture of cotton, there are obvious differences in the different fruit branch types. Both the growth status of cotton and its sensitivity to photoperiod have a significantly influence on flowering [9]. The fruit branch type of cotton changes the plant architecture and affects its flowering period. Therefore, genetic analysis on the regulation of the fruit branch type of cotton can provide a theoretical basis for selecting early-maturity cotton varieties with a compact plant architecture, concentrated flowering and a bolting period. In addition, the location of the genes that regulate the fruit branch can provide genetic resources for mechanized harvest cotton breeding.

BSA, which can locate the major genes of qualitative or quantitative traits based on the F_2_ population or RIL population, is a gene mapping method based on the linkage imbalance principle [10]. Using this method, DNA mixing pools were constructed using the DNA derived from the plants with extreme phenotypes of the target trait. Therefore, the DNA fragments related to the target traits were different in the two DNA mixing pools, and the genetic differences between the two mixing pools could achieve gene localization. This method has been widely used in cotton [11], Maize [12], rice [13], cucumber [14] and other crops. Candidate gene association analysis is a method to verify that a marker locus or a combination of markers is associated with a trait. Compared with genome-wide association analysis, candidate gene association analysis has more flexible testing methods [15]. Similarly, through candidate gene association analysis, an SNP located in exon 5 of the ZmGS3 gene was associated with maize grain length in different environments [16].

Previous studies have shown that cotton branches are controlled by a single gene located in the nucleus of the cell. The *GbNB* gene was reported to regulate the fruit branch types of cotton in Pima cotton, and the *GhNB* gene was reported to regulate the fruit branch types of upland cotton [5]. We also obtained the same result and detected a new allele in this study. Furthermore, through the candidate gene association analysis of *GhNB* or *GbNB* and the flowering period, the effect of the *GhNB* and *GbNB* genes on cotton flowering and the molecular markers related to early maturity in cotton were discovered.

## 2. Materials and Methods

### 2.1. Plant Materials

04N-11 was a nulliplex-branch mutant of upland cotton, Huaxin 102 was a normal branch variety of upland cotton, and Xinhai 18 was a nulliplex-branch variety of Pima cotton.

A total of 248 cotton varieties approved in Xinjiang, including 73 upland cotton early-maturity varieties; 79 medium-maturity varieties; 63 parts of Pima cotton varieties; 11 colored cotton varieties; 20 backbone parents and 2 self-breeding varieties, were collected to construct the Xinjiang natural population [17] (Supplementary Table S1).

### 2.2. Identification of Fruit Branch Types

The type of cotton fruit branch was divided into nulliplex branch, determinate branch and normal branch according to the boll position. Branches with cotton bolls growing directly in the axils of the main stem are called nulliplex branches; if the cotton has fruit branches but only one fruit node, with bolls clustered at the top of fruit branch, it is a determinate branch; and normal branches have more than one fruit node, and cotton bolls are borne on the top of fruit nodes.

### 2.3. BSA Sequence Analysis

BSA (Bulked Segregant Analysis) is a mixed grouping method, which uses a pair of parents with significant differences in traits to construct a separated population through crossbreeding, backcrossing and other methods. In this study, Huaxin 102 and 04N-11 were used as parents to construct an F_2_ population. A total of 30 randomly selected plants with nulliplex branches were collected from the population, and DNA was extracted and mixed equally to construct a nulliplex-branch DNA mixing pool. Additionally, 30 randomly selected plants with normal branches were collected from the population, and DNA was extracted and mixed equally to construct a normal-branch DNA mixing pool. Huaxin 102, 04N-11 and two DNA mixing pools were resequenced using an Illumina sequencing platform. The obtained sequencing data were compared with the reference genome of TM-1_ZJU_V2.1 (ftp://ftp.bioinfo.wsu.edu/species/Gossypium_hirsutum/ZJU_G.hirsutum_AD1genome_v2.1) (accessed on 1 May 2022) to identify SNP and InDel mutations between Huaxin 102 and 04N-11. The clean genomic data were generated and deposited in NCBI Sequence Read Archive (SRA) (https://www.ncbi.nlm.nih.gov/sra) under accession number PRJNA841236 (accessed on 1 May 2022). According to the SNP markers obtained by sequencing, the index value of each SNP marker in the two DNA mixing pools was calculated as follows:

$$\text{SNP-index}=\frac{SNP_{P-J}}{SNP_{P-J}+SNP_{P-Y}}.$$

(*SNP_P−J_* is the sequencing depth of the SNP marker genotype in 04N-11, and *SNP_P−Y_* is the sequencing depth of the SNP marker genotype in Huaxin 102).

### 2.4. Candidate Gene Sequencing and Allele Test

It has been reported that the *GbNB* gene in Pima cotton is allelic with the *GhNB* gene in upland cotton, both located on chromosome D07. In this experiment, we constructed an F_2_ genetic population with nulliplex-branch Xinhai 18 and 04N-11 as parents to verify whether the fruit branch type gene in 04N-11 was allelic with the *GbNB* gene of Xinhai 18. The *GhNB* gene in 04N-11 was cloned using TA cloning technology, to determine the type and number of mutations in the *GhNB* gene.

### 2.5. Identification of Fruit Branch Types and Investigation of Flowering Period

From 2018 to 2019, The materials of the Xinjiang natural population were planted in The Shihezi Academy of Agricultural Sciences in Xinjiang, for two consecutive years. The flowering period and fruit branch type of each material were investigated from mid-June to mid-July every year. According to the flowering period of each material (in which 50% of the plants had at least one open flower), the number of days from seeding emergence to flowering period was uniformly converted (Supplementary Table S1).

### 2.6. Association Analysis of Candidate Genes

The *GhNB* gene in upland cotton and the *GbNB* gene in island cotton were classified according to resequencing data from the Xinjiang natural population of 248 (Supplementary Table S2) [17]. The linkage interval of the *GhNB* or *GbNB* genes was determined according to the LD value, and SNP variation in the linkage interval was identified. Association analysis was performed at the candidate gene and haplotype level, with the flowering period as the phenotype.

## 3. Results

### 3.1. Phenotype Analysis of 04N-11 Nulliplex-Branch Mutant

**Figure 1:**
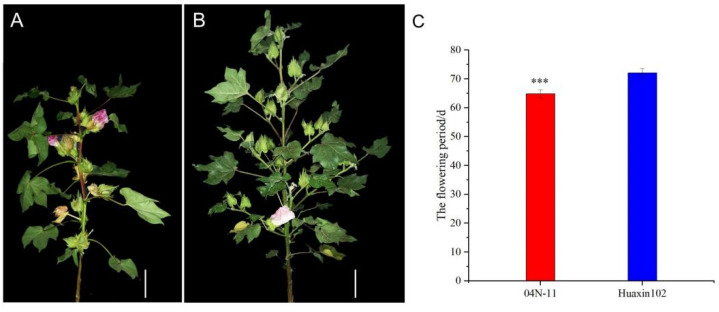
Fruit branch type and the flowering period of parent: (**A**) the original plant of 04N-11; (**B**) the original plant of Xinhai102. Bars = 10 cm in (**A**,**B**); (**C**): the flowering period of 04N-11 and Huaxin102 (we counted the number of days from seeding emergence to the flowering period). *** indicate significance at *p* < 0.001.

Phenotype analysis of 04N-11, which is a nulliplex-branch mutant of upland cotton, showed that cotton bolls were grown in the axils of the main stem, two bolls share a pair of sepals, short fruit branches are formed, and cotton bolls are clustered on the tops of the fruit branches (Figure 1A). Phenotype analysis of Huaxin 102, which is a normal branch of upland cotton, showed that the branches on the main stem include fruit branches and vegetative branches (Figure 1B). Fruit branches have multiple nodes, and each node is a growth unit bearing a boll and a leaf. Weak plants form only one unit per branch, with a boll at the top of each node. The axial primordium of the main stem leaf can be divided into two subsidiary tissues, which can be divided into a vegetative branch and a fruit branch; a nulliplex branch and vegetative branch; and two fruit branches, according to the plant growth. According to the statistics of the flowering periods of Huaxin 102 and 04N-11, 04N-11 flowers earlier than Huaxin 102 (Figure 1C). The flowering period of 04n-11 is about 6 to 7 days earlier than Huaxin102. The phenotypes of 04N-11 and Huaxin 102 were observed from 2018 to 2020, and the traits of branch type in 04N-11 and Huaxin 102 were stable and not affected by environmental conditions.

### 3.2. Nulliplex-Branch Traits Were Controlled by a Single Recessive Gene

The hybrid F_1_ population was derived from orthogonal and inverse crossing by Huaxin102 (normal branch) and 04N-11 (nulliplex branch). Phenotype analysis showed that all F_1_ plants were normal branch, indicating that the type of branch was a recessive trait controlled by nuclear genes. Then, the F_2_ population, derived from the self-crossing of the F_1_ population, were obtained; phenotype analysis showed that the 388 F_2_ plants contained 278 normal-branch plants and 110 nulliplex-branch plants in 2019, and the 1895 F_2_ plants contained 1455 normal-branch plants and 440 nulliplex-branch plants in 2020. Chi-square fitness test analysis showed that the separation ratio of normal branch and nulliplex branch was 3:1 in the F_2_ population (Table 1). These results suggest that the nulliplex-branch mutation of 04N-11 is controlled by a single recessive gene on the genome.

**Figure 2:**
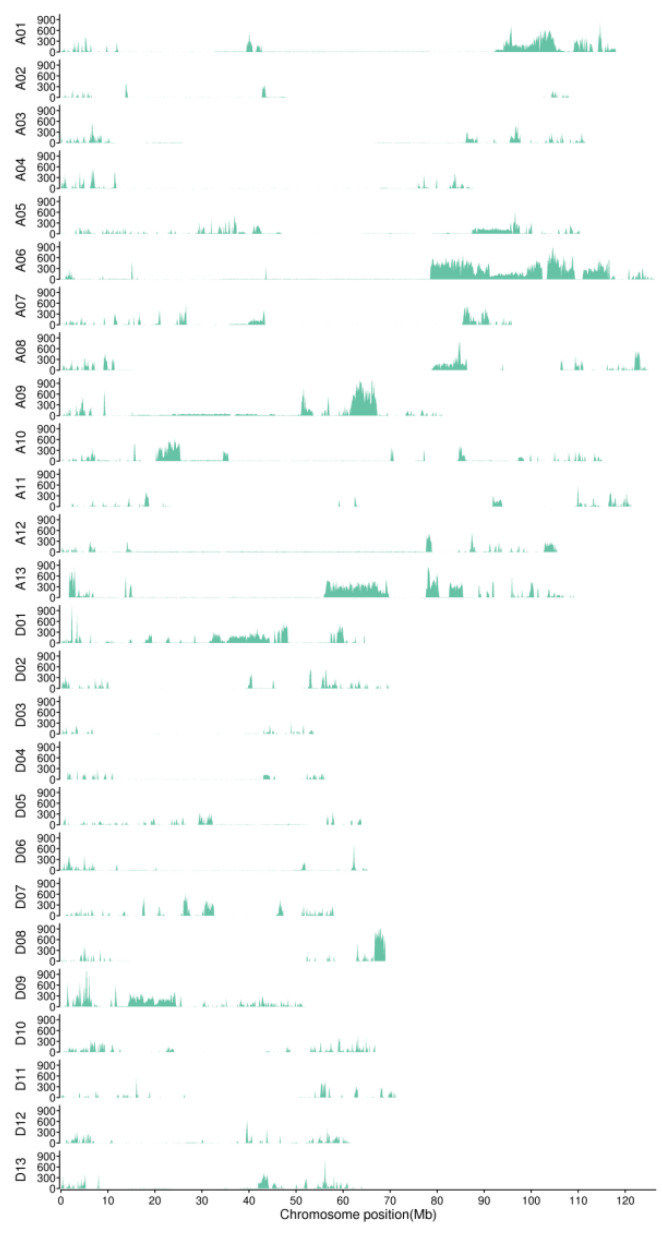
Distribution of SNP and InDel markers on each chromosome of huaxin 102 and 04N-11 genomes.

To map the genetic locus of the nulliplex branch, the BSA-seq method was performed on the F_2_ populations. The mixed pool was constructed using the extreme individuals of the normal branch and nulliplex branch of the F_2_ population, obtained by crossing Huaxin102 and 04N-11. In detail, 30 individual plants with the nulliplex branch and normal branch were equally mixed to obtain one DNA mixing pool marked as the J DNA mixing pool, and another marked as the Y DNA mixing pool. High-throughput genome sequencing was performed on two DNA mixing pool samples at 30×, and the two parents (huaxin 102 and 04N-11) at 10×. The obtained resequencing data were mapped to the reference genome of TM-1_ZJU_V2.1, then to identify SNP and InDel mutations between Huaxin 102 and 04N-11. Through comparative analysis, 815,493 SNPs and InDel variants, with polymorphism between Huaxin 102 and 04N-11, were screened from 730,630 SNP and 84,863 InDel variants. Analysis of the distribution of SNPs and InDels in the whole genome of Huaxin 102 and 04N-11 was performed, and showed aggregation in some genome segments (Figure 2).

**Figure 3:**
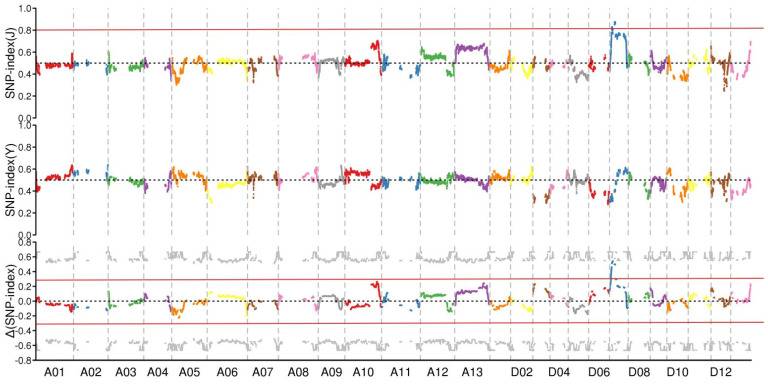
Δ(SNPs-index) values in the distribution of each chromosome.

The distribution of Δ(SNP-index) in the chromosomes of upland cotton was analyzed, and showed that the Δ(SNP-index) values of most of the SNP markers were between −0.3–0.3. Only Δ(SNP-index) values in the 12.16–14.82 Mb range on chromosome D07 exceeded 0.5, which suggests that this region is the candidate region of genes determining the fruit branch type of cotton (Figure 3).

### 3.3. Cloning and Allelic Test of 04N-11 Nulliplex-Branch Candidate Gene

**Figure 4:**
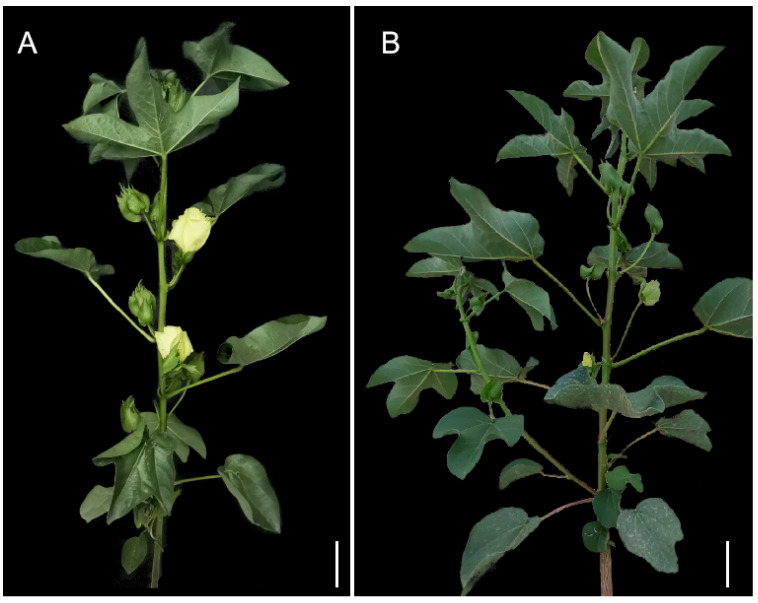
The plant architecture of Xinhai 18 and the F_1_ plant: (**A**) The original plant of Xinhai18; (**B**) the original plant of F_1_; bars = 10 cm in (**A**,**B**).

In this study, phenotype identification confirmed that the nulliplex-branch trait in 04N-11 was controlled by a single recessive gene on the genome, and a candidate region for the cotton fruit branch type was located on the D07 chromosome at 12.16~14.82 Mb using BSA-seq. The *GhNB* gene at 15.1Mb on the right of the candidate region was reported to control the fruit branch type of cotton [18]. The *GbNB* gene at 15.4Mb on chromosome D07 was reported to control the type of cotton fruit branch in Pima cotton, and the *GbNB* and *GhNB* genes were allelic [5]. In order to verify whether the *GhNB* or *GbNB* gene is allelic with the gene determining the fruit branch type of 04N-11, a nulliplex-branch Pima cotton Xinhai 18 (the nulliplex branch was determined by *GbNB*) and 04N-11 were crossed to observe the progeny phenotype (Figure 4A). In 2019 and 2020, the fruit branch type of individual plants of the F_1_ and F_2_ populations were investigated, respectively. Additionally, it was shown that the cotton bolls of the F_1_ and F_2_ populations were directly grown in the axils of the main stem or clustered on the tops of fruit branches, and the fruit branch types were nulliplex (Figure 4B).

**Figure 5:**
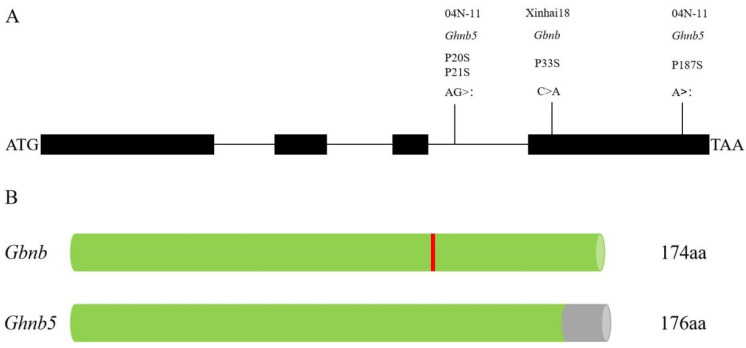
*Gbnb* and *Ghnb5* gene mutation sites in Xinhai 18 and 04N-11 and protein sequence structure: (**A**) gene mutation site; (**B**) schematic diagram of protein sequence (green cylinders: native amino acid; gray cylinder: amino acid sequence changes caused by frameshift mutation; red vertical lines: amino acid changes).

The results indicate that the gene controlling fruit branch type in 04N-11 was allelic with the *GbNB* gene in Xinhai 18. Therefore, the *GhNB* gene in 04N-11 was cloned to determine whether the gene was mutated. The *GhNB* gene sequence on D07 in 04N-11 was obtained, and showed that there were three deletion bases of this gene in the nulliplex-branch material 04N-11—namely, the deletion of the A base located in the fourth exon P187, the deletion of the A base located in the third intron P20, and the deletion of the G base located in the third intron P21 (Figure 5A,B). The deletion of base A in the fourth exon causes frameshift mutation and changes the sequence of amino acids and the structure of the protein. A new *GhNB* recessive mutant genotype, *Ghnb5*, was obtained in 04N-11.

### 3.4. Association Analysis of Candidate Genes

The *GhNB* and *GbNB* genes belong to the *TFL1* subfamily of the *PEBP* gene family, which is related to flower development in plants. After a photoperiod induction, the expression products of *FT* and *TFL1* of the *PEBP* gene family were combined with the FD protein to promote or inhibit flowering, and showed a quantitative effect [5]. In this study, 248 Xinjiang cotton varieties, including upland cotton, Pima cotton and colored cotton, were planted in The Shihezi Agricultural Research Institute of Xinjiang for two consecutive years from 2018 to 2019. The flowering period and fruit branch type (nulliplex branch or normal branch) of each material were investigated. According to the number of days from seeding emergence to flowering, a total of 236 materials over two years of flowering were obtained.

**Figure 6:**
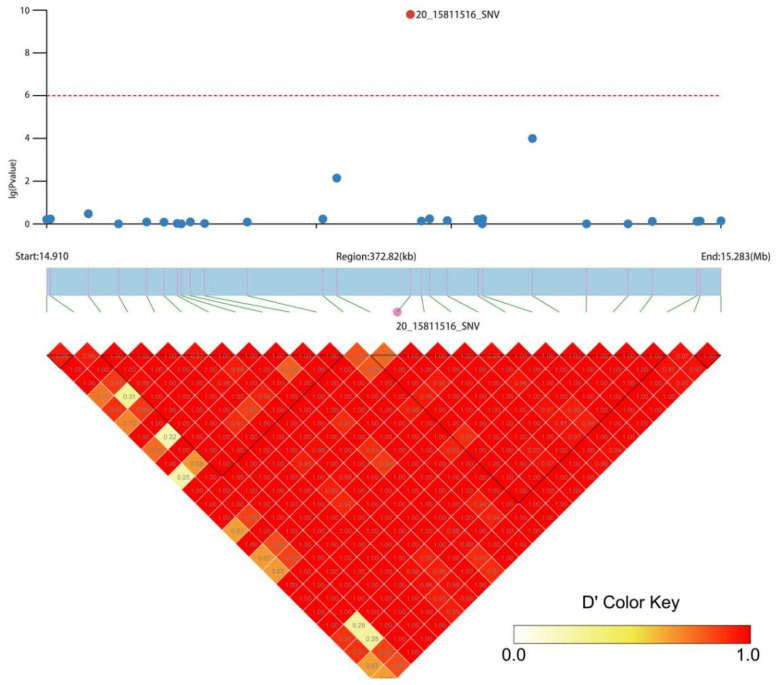
Association analysis of candidate genes at haplotype level.

The linkage interval of the *GhNB* or *GbNB* genes was screened according to the LD values of the molecular markers near the *GhNB* or *GbNB* genes. The interval length was 372.82 Kb, and there were 26 molecular markers. Then, 26 polymorphic molecular markers were used for association analysis with the flowering period (we counted the number of days from seeding emergence to the flowering period time) at the haplotype level, and only one SNP marker, 20_15811516_SNV, was significantly correlated with the flowering period in this linkage interval (Figure 6).

**Figure 7:**
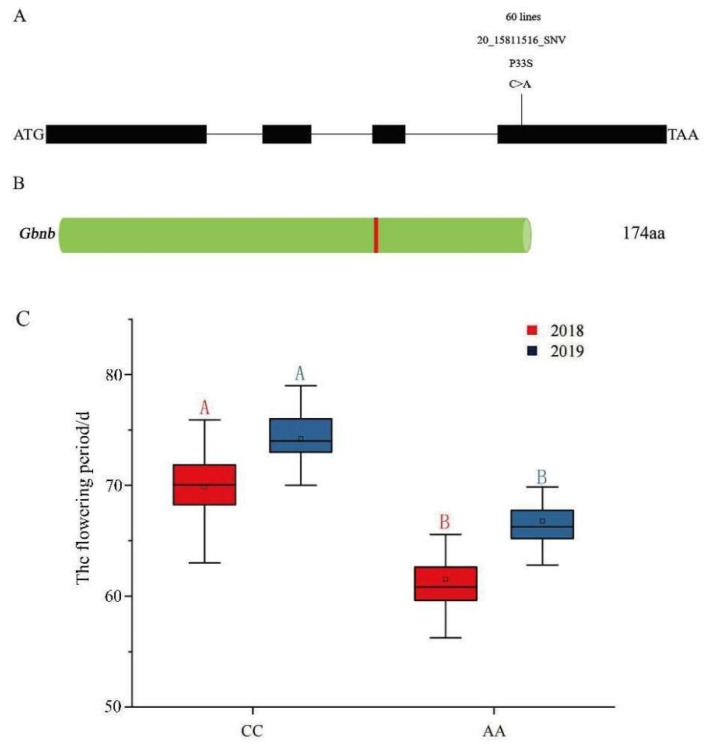
Analysis of gene variation and haplotype of the significant SNP: (**A**) gene mutation site; (**B**) schematic diagram of protein sequence (green cylinders: native amino acid; red vertical lines: amino acid changes); (**C**) association analysis of candidate genes (A and B represent extremely significant differences between the two groups, *p* < 0.01).

The marker 20_15811516_SNV is located with the fourth exon of the *GhNB* or *GbNB* genes, and the mutation of the 33rd base leads to the change in the encoded amino acid from proline to serine, resulting in a change in gene function (Figure 7A,B). Two genotypes were obtained from the 236 materials, including 176 wild-type materials (CC) and 60 mutant materials (AA) (Table S2). The Duncan method was used to conduct a T-test on the flowering period (we counted the number of days from seeding emergence to the flowering period) of the two types of materials. The difference between the two genotypes was extremely significant, and the flowering period of the mutated materials was significantly earlier (Figure 7C).

The fruit branch types of 236 materials were identified. Among the materials labeled 20_15811516_SNV genotype CC, 173 materials are normal branch and 3 materials are nulliplex branch. In genotype AA, 3 materials are normal branch and 57 materials are nulliplex branch. An independence test of the genotype of the marker and the fruit branch type of the cotton was conducted (χ^2^ = 205.39 ($\chi_{0.01}^{2}$ = 6.63, *p* = 0.01)) and the marker was significantly correlated with fruit branch type and the flowering period of cotton (Table 2).

## 4. Discussion

The plant architecture of cotton is determined by its fruit branch type, internode length and fruit branch angle, among which fruit branch type is the most important factor affecting plant architecture. The common fruit branch types are the nulliplex branch, determinate branch and normal branch. The number of leaves on the fruit branches was equal to the number of fruit nodes, and the types of fruit branches and the number of fruit branches affected the number of fruit nodes. Compared with the normal branch, the number of plant leaves of the nulliplex-branch varieties was significantly less than that of the normal branch. On normal-branch cotton varieties, each fruit branch will form 2–3 fruit nodes, and generally, there will be 10 effective fruit branches. On the nulliplex branch, cotton bolls are in the axils of the main stem or clustered at the top of the fruit branch. In general, there was no significant difference in the number of bolls between the nulliplex branch and normal branch. However, compared with nulliplex branch, normal-branch varieties had a higher, more stable yield. Cotton shaped under high-yield cultivation mode in Xinjiang is characterized by short plants, a high planting density and a short growth period [19]. Therefore, the study of the genetic mode of fruit branched is beneficial in determining plant architecture in the breeding of cotton. In this study, one gene, which play an important role in determining the branch-type of cotton, was confirmed by constructing a cotton F_2_ population derived from the different fruit branch varieties or lines.

In this study, the whole genomes of Huaxin 102 and 04N-11 were sequenced, and they were mapped to the reference genome to identify the polymorphism SNP and InDel. However, the molecular markers identified are unevenly distributed throughout the genome, due to the different genetic backgrounds between the two parents. The coverage and depth of the gene sequencing obtained through quality control also affect the identification of molecular markers (Figures S1 and S2). The candidate intervals of the target gene were obtained at an interval of 12.16–14.82 Mb on chromosome D07. Additionally, recent studies showed that a *GhNB* gene at 15.1Mb on the right of this region was involved in the regulation of the cotton fruit branch [20], and the homologous gene *GbNB* in Pima cotton controlled the branch type of Pima cotton [18]. The *GbNB* gene is located at 15.4 Mb on chromosome D07 and is allelic to the *GhNB* gene. By crossing Pima cotton and upland cotton cultivars, all F_2_ populations’ phenotypes of fruit branch are nulliplex [5]. In this study, the fruit branch of the F_2_ population derived from 04N-11 crossed with Xinhai 18 were all nulliplex (Figure 4B). Combined with the location results of BSA-seq, this indicates that the gene controlling the types of cotton fruit branches in 04N-11 was the *GhNB* gene. In this study, only 14 molecular markers, including 12 SNP markers and 2 InDel markers, were identified at the 14.9–17.2 Mb interval of the D07 chromosome. Fewer molecular markers in this region may be the main reason affecting the localization interval. Sequencing the *GhNB* gene in 04N-11 showed that the *Ghnb5* gene in 04N-11 had three deletion bases.

The plant’s response to photoperiod is transmitted to *FT*/*TFL1* via the *CO* gene. The *FT*/*TFL1* protein, a production of *FT*/*TFL1* gene expression, competitively binds the FD protein to promote or inhibit flowering [21]. The *GhNB* and *GbNB* genes belong to the *TFL1* gene, and their function is to inhibit plant flowering. The mutations of the *GhNB* or *GbNB* genes in cotton affected the plant architecture and the flowering period of cotton. In 236 materials, the flowering period of *GhNB* or *GbNB* mutated materials was significantly earlier than in the wild variety, and the phenotype of mutated materials was mainly the nulliplex branch. At the haplotype level, SNP marker 20_15811516_SNV in the *GhNB* and *GbNB* genes were significantly correlated with flowering stage. These results indicate that the *GhNB* and *GbNB* gene were involved in the regulation of plant type and flowering stage of cotton. The difference in flowering stage between the nulliplex branch and normal branch was more than 10 days, indicating that cotton flowering was regulated by multiple pathways simultaneously. Therefore, it is possible to select suitable flowering varieties based on the plant architecture of nulliplex branch or normal branch.

**Figure 8:**
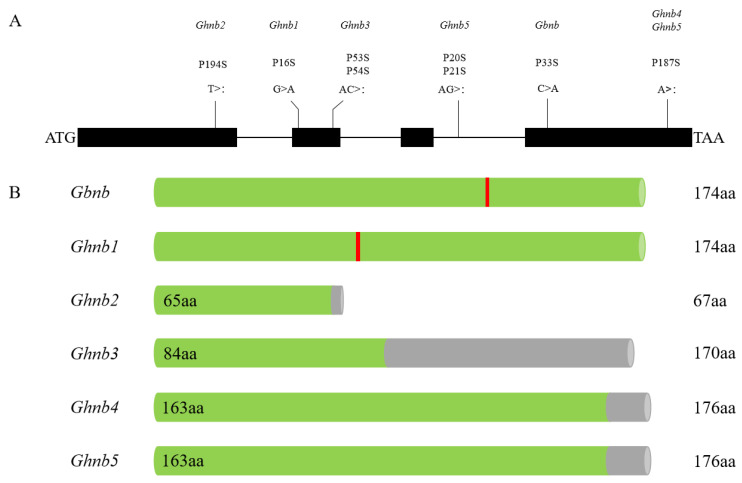
Mutation types and proteins of *GhNB* and *GbNB* genes: (**A**) gene mutation site; (**B**) schematic diagram of protein sequence (green cylinders: native amino acid; gray cylinder: amino acid sequence changes caused by frameshift mutation; red vertical lines: amino acid changes).

There were four recessive mutant genotypes of *GhNB* previously reported in upland cotton, while only one recessive mutant genotype of *GbNB* was reported in Pima cotton (Figure 8), and the genotype of the *GhNB* gene was more abundant in upland cotton [5]. Mapping and analyzing the genetic basis of cotton fruit branch types can provide genetic resources and a theoretical basis for the breeding of compact and early-maturity cotton varieties, which is suitable for machine picking. The two InDel markers; the deletion of the A base located in the fourth exon P187; the deletion of the A base located in the third intron P20; and the deletion of the G base located in the third intron P21 in 04N-11 and the SNP marker 20_15811516_SNV obtained by association analysis can be used as co-separation markers for molecular-assisted design breeding [22] or variety identification [23].

## 5. Conclusions

A candidate interval of 12.16–14.82 Mb on chromosome D07 was obtained in this study. The F_2_ population constructed by Xinhai 18 and 04N-11 as parents were all nulliplex branch. These results indicated that *GhNB* was a candidate gene of nulliplex branch in upland cotton, and the recessive mutant genotype in 04N-11 was *Ghnb5*. There were two InDel markers in 04N-11 *GhNB*, which could be used as co-separation markers for molecular-assisted design breeding or for variety identification. Through candidate gene association analysis, an SNP marker (20_15811516_SNV) associated with early maturity was discovered in the Xinjiang natural population.

## Figures and Tables

**Table 1:** Statistical table of normal branch and nulliplex branch of F_2_ population.

Year	Normal Branch	Nulliplex Branch	χ^2^
2019	278	110	1.98
2020	1455	440	3.27

**Table 2:** Statistics of cotton fruit branch types and different genotypes.

	Normal Branch	Nulliplex Branch
CC	173	3
AA	3	57

## Data Availability

The data presented in this study are openly available in NCBI Sequence Read Archive at https://www.ncbi.nlm.nih.gov/sra (accessed on 1 May 2022), reference number PRJNA841236.

## Supplementary Materials

The following supporting information can be downloaded at: https://www.mdpi.com/article/10.3390/plants11111483/s1, **Supplementary Table S1**. The flowering period and the type of branch of every material; **Supplementary Table S2**. The genotype of 20_15811516_SNV in GhNB or GbNB genes in the Xinjiang natural population; **Supplementary Figure S1**. Genome coverage and coverage depth distribution of 04N-11 se-quencing data; **Supplementary Figure S2**. Genome coverage and coverage depth distribution of Huaxin102 sequencing data.
