# Supplementary figures and images for: Genetic Mapping and Analysis of a Compact Plant Architecture and Precocious Mutant in Upland Cotton
Source: Plants (Basel). 2022 May 31;11(11):1483. doi: 10.3390/plants11111483 (PMC9182648; doi:10.3390/plants11111483)

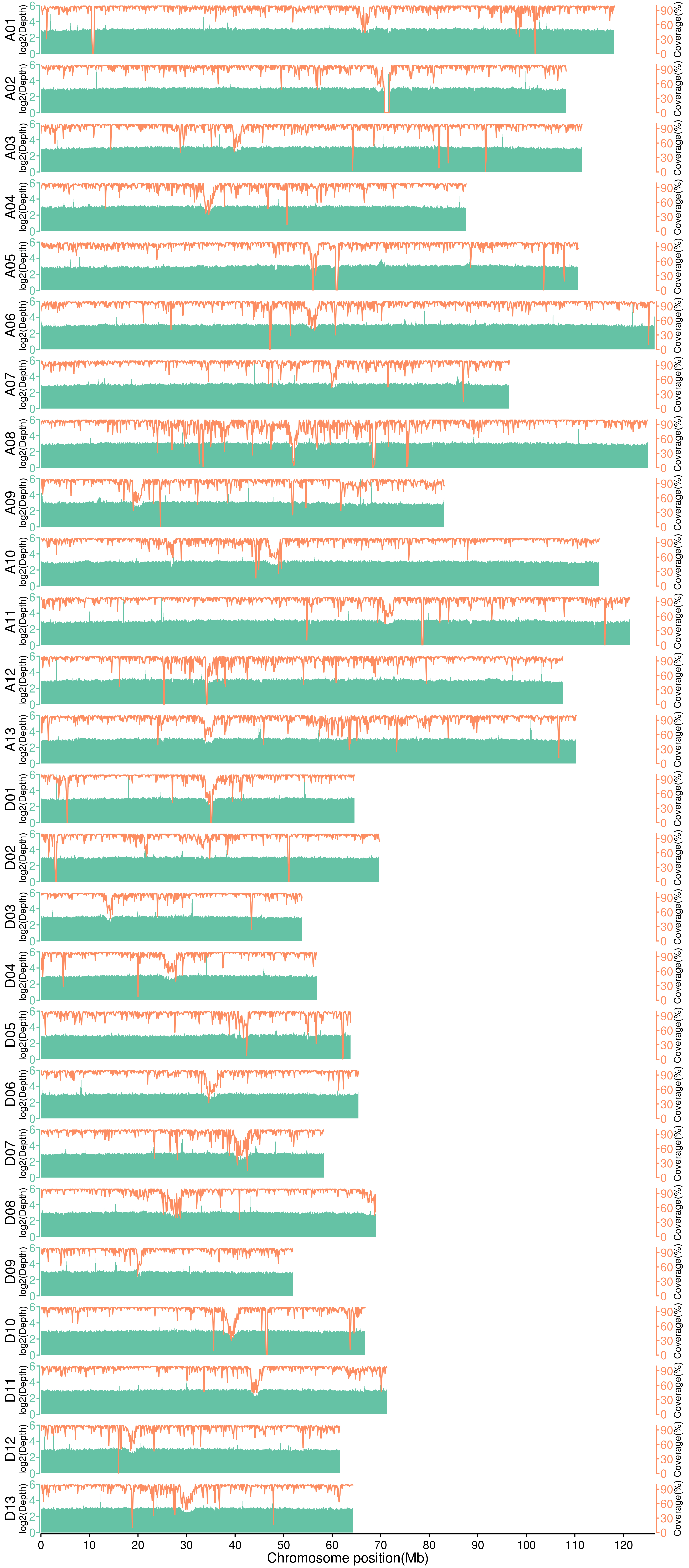

Supplement: Supplementary file 1 [file plants-11-01483-s001.zip › Figure S1.jpg]

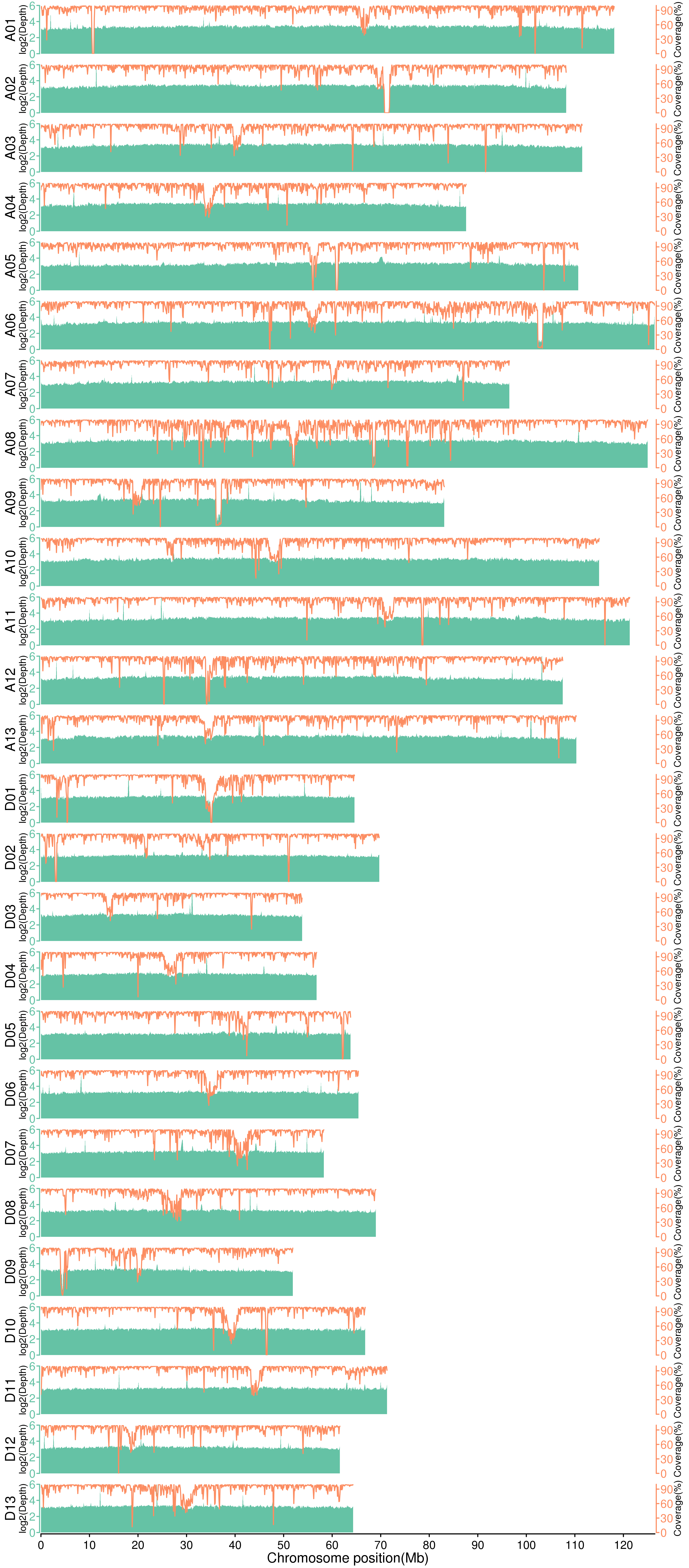

Supplement: Supplementary file 1 [file plants-11-01483-s001.zip › Figure S2.jpg]
